# Oridonin prevents oxidative stress‐induced endothelial injury via promoting Nrf‐2 pathway in ischaemic stroke

**DOI:** 10.1111/jcmm.16923

**Published:** 2021-09-12

**Authors:** Lei Li, Shu‐Qi Cheng, Wei Guo, Zhen‐Yu Cai, Yu‐Qin Sun, Xin‐Xin Huang, Jin Yang, Juan Ji, Ya‐Yun Chen, Yin‐Feng Dong, Hong Cheng, Xiu‐Lan Sun

**Affiliations:** ^1^ Neuroprotective Drug Discovery Key Laboratory Jiangsu Key Laboratory of Neurodegeneration Nanjing Medical University Nanjing China; ^2^ The First Affiliated Hospital of Nanjing Medical University Nanjing China; ^3^ Nanjing University of Chinese Medicine the Affiliated Hospital of Nanjing University of Chinese Medicine Nanjing China

**Keywords:** endothelial cell, ischaemic stroke, Nrf‐2, oridonin, oxidative stress

## Abstract

Oridonin, a natural diterpenoid compound extracted from a Chinese herb, has been proved to exert anti‐oxidative stress effects in various disease models. The aim of the present study was to investigate the protective effects of oridonin on oxidative stress‐induced endothelial injury in ischaemic stroke. We found oridonin repaired blood‐brain barrier (BBB) integrity presented with upregulation of tight junction proteins (TJ proteins) expression, inhibited the infiltration of periphery inflammatory cells and neuroinflammation and thereby reduced infarct volume in ischaemic stroke mice. Furthermore, our results showed that oridonin could protect against oxidative stress‐induced endothelial injury via promoting nuclear translocation of nuclear factor‐erythroid 2 related factor 2 (Nrf‐2). The specific mechanism could be the activation of AKT(Ser473)/GSK3β(Ser9)/Fyn signalling pathway. Our findings revealed the therapeutic effect and mechanism of oridonin in ischaemic stroke, which provided fundamental evidence for developing the extracted compound of Chinese herbal medicine into an innovative drug for ischaemic stroke treatment.

## 1 INTRODUCTION

Stroke is the second leading cause of global mortality and the major cause of neurological disability.^1^ At present, thrombolysis and thrombectomy are the main treatments for ischaemic stroke.^2^ However, current effective therapies are limited to a small percentage of people due to the narrow treatment time window and secondary injuries of haemorrhage transformation.^3^ Therefore, novel effective therapeutic targets and drugs are urgent to be discovered for ischaemic stroke in addition to thrombolytic treatment.

Blood‐brain barrier plays a vital role in regulating the exchange of various substances and cells at the blood‐brain interface between periphery and central nervous system (CNS).^4^ After ischaemic stroke, the serious disruption of BBB and the infiltration of plenty of peripheral immune cells increase the occurrence of neuroinflammation, and thus aggravate brain infarction.^5^ The brain vascular endothelial cells are the major component of BBB, whose intercellular junction forms high trans‐endothelial electrical resistance and low permeability.^6^, ^7^, ^8^ Reportedly, the overloaded oxidative stress reactions severely cause endothelial cells apoptosis and decrease TJ proteins expression level, and thereby increase BBB permeability after ischaemic stroke.^9^, ^10^ Thus, alleviating the endothelial cells injuries from excessive oxidative stress reactions could be a promising strategy for ischaemic stroke treatment.

Oridonin, a natural diterpenoid compound extracted from a Chinese herb Rabdosia rubescens and a regulator of the AKT signalling pathway,^11^, ^12^ exhibits numerous biological activities and effects, including anti‐oxidative stress and anti‐inflammatory activity in animal models of LPS‐induced acute lung injury and cardiac hypertrophy.^13^, ^14^, ^15^ However, whether oridonin can ameliorate oxidative stress in endothelial cells and improve the integrity of BBB after ischaemic stroke remains unclear. Therefore, the aim of the present study was to investigate the effects and the involved mechanisms of oridonin in ischaemic stroke model.

## 2 MATERIALS AND METHODS

### 2.1 Experimental animals and administration

C57BL/6N male mice weighing 20 ± 2 g were obtained from the Animal Resource Center of the Faculty of Medicine, Nanjing Medical University for the experiment. All animals operating procedures were carried out following the regulations of the Animal Protection and Use Committee of Jiangsu Experimental Animal Association and approved by the Animal Protection and Ethics Committee (IACUC) of Nanjing Medical University. The mice were given a week of environmental adaptation before the experiment. All animals were randomly divided into the following three groups by a randomized block design: sham + vehicle group, tMCAO + vehicle group and tMCAO + oridonin group. For sham group, mice were subjected to the same procedures as the other groups excepting for inserting filament. In the tMCAO +oridonin group, the mice were intraperitoneally administrated with oridonin once per day at a dose of 20 mg/kg for three consecutive days after reperfusion.

### 2.2 Reagents and drugs

Oridonin (28957‐04‐2, TargetMol, Shanghai, China) was dissolved in dimethyl sulfoxide (DMSO) for storage (500 mM) and then further diluted to working concentrations. Same volume or amount of DMSO was applied as vehicle. In vitro experiments, stock solutions of oridonin were further diluted to different working concentrations with culture medium, and serial concentrations of oridonin from 1 μM to 10 μM were not toxic for the cellular activity in our experiment.

### 2.3 Preparation for transient focal cerebral ischaemia and reperfusion model

The protocol of transient middle cerebral artery occlusion (tMCAO) was conducted by using the intraluminal filament technique as previously described.^16^ Briefly, the common carotid artery (CCA), external carotid artery (ECA) and internal carotid artery (ICA) were exposed by surgical operation from midline neck incision carefully. The CCA was temporarily closed and nylon monofilament (0.18 ± 0.01 mm, L1800, Guangzhou Jialing Biotechnology, Guangzhou, China) was inserted into the ICA through the ECA until it reached the middle cerebral artery, and it was left for 45 minutes. The nylon monofilament was pulled out from the ICA to achieve reperfusion after 45 minutes occlusion. To ensure the successful establishment of transient focal cerebral ischaemia and reperfusion model, local cerebral blood flow (LCBF) was measured using the MoorFLPI Full‐field Laser Perfusion Imager (MoorFLPI‐2, Gene&I, Beijing, China). From the beginning of reperfusion, oridonin was administrated (i.p. 20 mg/kg/d) once a day for consecutive 3 days.

### 2.4 Behavioural assessment

Neurological deficit scoring was performed after reperfusion using modified Longa score.^16^ It was graded on a scale of 0 to 5: 0 = no neurological deficits, 1 = failure to extend left forepaw fully, 2 = circles to the left, 3 = falls to the left, 4 = no spontaneous walking with loss of consciousness and 5 = dead. The scoring procedure was performed by the investigator who was blinded to each mouse. Beam walking test was performed to assess dynamic balance impairments and mobility disability in neurological disorders.^17^ During the beam walking test, the wooden beam with 2.5 × 180 cm size was placed at a height of 1 m above the floor, and the mice behaviour was detected by crossing the beam, and the times of foot slip off the top surface and the duration of walking on the beam were recorded. Each test of mice was conducted by two times in forward direction and another two times in reverse direction. The results of four times tests were averaged. All beam walking tests were conducted 3 days after ischaemia.

### 2.5 Determination of the infarct volume

The infarct volume assessment by 2, 3, 5‐Triphenyltetrazolium chloride (TTC) staining was performed to histologically verify the success of the model.^18^ The mice were anaesthetized and sacrificed after 3 days after surgery. The brains were quickly taken out and slipped by 2 mm thickness. TTC (T8877‐25g, Sigma Aldrich, Shanghai, China) was dissolved into PBS (1%). The slices were incubated in the TTC solution for staining for 15 minutes at 37℃ and infused in 4% paraformaldehyde for fixation overnight. The photographs were taken, and the infarct volumes were measured and analysed by Image J software (version1.8.0, National Institutes of Health, Bethesda, USA).

### 2.6 Evans blue dye leakage analysis

Destruction of the BBB was analysed by Evans blue (EB) assay 3 days after tMCAO. The mice were subjected to 2% EB dye (YE8010, YIFEIXUE BIO TECH, Nanjing, China) dissolved in normal saline intravenously and kept circulation for 2 hours, and the mice were anaesthetized and perfused with PBS until the blood was clarified. The right brain was weighted and homogenized in formamide (1:20 w/v). The homogenates were incubated at 60 ℃ for 40 hours so that EB could resolve into the formamide and then centrifuged at 16000g for 30 minutes. Taking 200 μL supernatant to the 96‐well plates and the amount extravasated amount of EB was quantified by spectrophotometer at 620 nm (BioTek instrument, EL×800, USA).

### 2.7 Immunohistochemistry staining

The mice were perfused with PBS (G4202, Servicebio, Wuhan, China) and 4% paraformaldehyde (30525‐89‐4, Nanjing Chemical Reagent, Nanjing, China). After dehydration, permeation and embeddedness, the brain tissues were sliced by 10 μM thickness and placed in the same position of the six glass slides, and three brain slides are pasted on each glass slide. Next, dewaxing and hydration were carried out. The slices were blocked in 10% goat serum / PBST at room temperature and incubated with primary antibodies overnight at 4℃. The dilutions of primary antibodies were used in the experiments included as follows: NeuN (1:500, ab177487, Abcam, Cambridge, MA, USA), Iba1 (1:500, NB100‐1028, Novus, Centennial, Colorado, USA), CD45 (1:200, ab40763, Abcam, Cambridge, MA, USA), Ly6G (1:200, #31469, CST, Boston, USA), IB4 (1:200, I21414, Invitrogen, Carlsbad, CA, USA), NF‐κB (p65) (1:200, ab32536, Abcam, Cambridge, MA, USA), Nrf‐2 (1:200, 16396–1‐AP, Proteintech, Chicago, USA) and Fyn (1:300, ab125016, Abcam, Cambridge, MA, USA). The slices were washed by PBST and incubated with secondary antibodies (1:1000 dilution, Invitrogen, Carlsbad, CA, USA) for 1h at room temperature. DAPI (C3619‐PI19B, Southern Biotech, Birmingham, AL, USA) was incubated for 10 minutes. The quantification of images was analysed with Image J software as previously described.

### 2.8 Quantitative real‐time PCR

The total RNA was extracted by using the TRIzol reagent (Invitrogen Life Technologies, CA, USA) according to the manufacturer's instructions. The concentration and purity of the total RNA were measured using a NanoDrop 2000 spectrophotometer (NanoDrop Technologies, Thermo Scientific, Waltham, Massachusetts, USA). A reverse transcription kit was used to synthesize the complementary DNA. Reverse transcription with RNA was conducted using SuperScript III Reverse Transcriptase (Invitrogen, Carlsbad, CA, USA), and PCR was performed using QuantStudio 5 Real‐Time PCR System (Applied Biosystems, NY, USA). Quantitative PCR was performed using SYBR Green Master Mix and the Bio‐rad CFX‐96 real‐time PCR instrument. The amplification specificity was validated by the presence of a single peak in the melting curves. In all independent experiments, GAPDH was used as an internal control, and the relative expression of the target genes was determined using the 2^(−ΔΔCT)^ method. Primer sequences for RT‐PCR were listed in Table 1.

**TABLE 1:** Gene primer sequence

Gene symbol	Primer sequence (5^,^ −3^,^)
CXCL‐1 F	CTGGGATTCACCTCAAGAACATC
CXCL‐1 R	CAGGGTCAAGGCAAGCCTC
CLCL‐3 F	TCCCCCATGGTTCAGAAAATC
CXCL‐3 R	GGTGCTCCCCTTGTTCAGTATCT
CXCL‐12 F	TGCATCAGTGACGGTAAACCA
CXCL‐12 R	TTCTTCAGCCGTGCAACAATC
IL‐1β F	GCAACTGTTCCTGAACTCAACT
IL‐1β R	ATCTTTTGGGGTCCGTCAACT
TNF‐αF	GACGTGGAACTGGCAGAAGAG
TNF‐α R	TTGGTGGTTTGTGAGTGTGAG
IL‐6 F	CCAAGAGGTGAGTGCTTCCC
IL‐6 R	CTGTTGTTCAGACTCTCTCCCT
IFN‐γ F	ATGAACGCTACACACTGCATC
IFN‐γ R	CCATCCTTTTGCCAGTTCCTC
MCP‐1 F	TTAAAAACCTGGATCGGAACCAA
MCP‐1 R	GCATTAGCTTCAGATTTACGGGT
IL‐10 F	GCTCTTACTGACTGGCATGAG
IL‐10 R	CGCAGCTCTAGGAGCATGTG
Arg‐1 F	CTCCAAGCCAAAGTCCTTAGAG
Arg‐1 R	AGGAGCTGTCATTAGGGACATC
TGF‐β F	CTCCCGTGGCTTCTAGTGC
TGF‐β R	GCCTTAGTTTGGACAGGATCTG

### 2.9 Oxygen‐glucose deprivation and reoxygenation

The oxygen‐glucose deprivation and reoxygenation (ODG/R) was performed as described previously.^19^ In briefly, before the OGD experiment, bEND.3 cells were cultured with DMEM complete medium (C11995500BT, Gibco, Grand Island, NY, USA) in an incubator (Thermo Fisher Scientific, Waltham, MA, USA) with 95% air and 5% CO2 at 37°C. During the OGD procedure, cells were treated with deoxygenated condition (95% N_2_ and 5% CO_2_) and glucose‐free DMEM (Life Technologies, 11966‐025, Gaithersburg, MD, USA) for 6 hours. After OGD, the cells were transferred to a normoxic incubator and replaced with normal DMEM medium.

### 2.10 Cell culture and drug treatment

Mouse brain microvascular endothelial cells bEND.3 were selected in vitro. The bEND.3 cells were cultured with DMEM medium (C11995500BT, Gibco, Grand Island, NY, USA), added with 15% FBS (10099141, Gibco, Grand Island, NY, USA) and 1% penicillin/streptomycin in an incubator (95% air and 5% CO_2_) at 37°C. The cells were seeded into 24‐well plates before exposure to OGD treatment for 6 h. When the reperfusion was performed, oridonin was added into the normal DMEM medium simultaneously. After OGD/R and drug treatment, the biochemical indicators were measured and subsequent processes were carried out.

### 2.11 Endothelial cell monolayer permeability assay

The BBB function was simulated in vitro by endothelial cell monolayer permeability assay. The endothelial monolayer permeability to neutrophils (inflammatory cells) was detected by Anopore membrane 24‐well cell culture inserts with 5.0 μm pore size (0801040, Corning, NY, USA).^20^ Endothelial cells were placed on the upper side of the insert and allowed to grow to confluence. After the reperfusion for 12 hours, the neutrophils were added into the endothelial monolayer. Neutrophils infiltrating through the inserts to the bottom of the 24‐well plates were fixed for further immunofluorescence staining after the next 12 hours.

### 2.12 Flow cytometry analysis

bEND.3 cell apoptosis was measured by an Annexin V‐FITC apoptosis analysis kit (KGA102, KeyGEN BioTECH, Nanjing, China). The cells were collected and washed with PBS. All procedures were performed by the manufacturer's instructions using flow cytometer analysis (BD FACSAria II, NY, USA). A minimum of 10,000 events were read.

### 2.13 Immunocytochemistry staining

Fixed cells with 4% paraformaldehyde and blocked with 3% BSA solution containing 0.1% triton for 1h at room temperature. Primary antibodies were incubated in 4℃ overnight, including Ly6G (1:200, #31469, CST, Danvers, MA, USA), NF‐κB (1:200, ab32536, Abcam, Cambridge, MA, USA), Nrf‐2 (1:200, 16396–1‐AP, Proteintech, Chicago, USA) and Fyn (1:300, ab125016, Abcam, Cambridge, MA, USA), and the samples were washed for three times. The fluorescence second antibodies (1:1000 dilution, Invitrogen, Carlsbad, CA, USA) were incubated for 1h at room temperature. Cell nucleus was stained with DAPI for 20 minutes. Images were taken using a confocal microscope (Zeiss LSM710, Jena, Germany) and analysed and quantified by using Software Image J (Version 1.8.0, National Institutes of Health, Bethesda, USA).

### 2.14 Reactive oxygen species measurement

Brain reactive oxygen species (ROS) level measurement was performed by using ELISA kit with by double‐antibody sandwich method (TIFEIXUE BIO TECH, Nanjing, China), according to the manufacturer's instructions. Briefly, the purified mouse ROS antibody was coated with the microporous plate. The right brain supernatant was successively added and then combined with the HRP‐labelled ROS antibody to form the antibody‐antigen‐enzymatic antibody complex, then thoroughly washed and colorated with TMB. The concentration of ROS in the sample was calculated by the standard curve and the absorbance (OD) of the sample, measured at 450 nm with a microplate analyzer.

Intracellular ROS levels were measured with CellRoX Green Reagent (Maokang Biotechnology, Shanghai, China) according to the manufacturer's instructions. Briefly, CellRox Green Reagent (5 μM) was added to each well for 30 minutes at 37℃. Then, the cells were washed three times with PBS. Images were taken using a confocal microscope (Zeiss LSM710, Jena, Germany) and quantified by Software Image J.

### 2.15 Western blotting

Protein lysates were obtained by using RIPA lysis buffer (Beyotime Biotechnology, Shanghai, China) and quantified by BCA method according to the manufacturer's instructions (Beyotime Biotechnology, Shanghai, China). The protein electrophoresis was conducted by SDS‐PAGE, and proteins were transferred to PVDF membranes (Roche, Mannheim, Germany). The PVDF membranes were blocked with 5% BSA/TBST at room temperature for 2 hours and incubated with primary antibodies for 12 hours at 4℃. After washing the membranes three times by TBST, HRP‐conjugated secondary antibodies incubation was conducted for 2 hours. The protein bands were captured by ECL method and Tanon 5200 Chemiluminescence image analysis system (Tanon, Shanghai, China). The proteins grayscales were quantified by Image J software. The primary antibodies used in this experiment including as follows: ZO‐1 (1:1000, 21773–1‐AP, Proteintech, Chicago, USA), occludin (1:1000, 66378‐1‐lg, Proteintech, Chicago, USA), claudin‐5 (1:1000, ab131259, Abcam, Cambridge, MA, USA), NF‐κB (p65) (1:1000, ab32536, Abcam, Cambridge, MA, USA), Bax (1:1000, 60267‐1‐lg, Proteintech, Chicago, USA), Bcl‐2 (1:1000, ab182858, Abcam, Cambridge, MA, USA), haem oxygenase‐1 (HO‐1, 1:1000, 27282‐1‐AP, Proteintech, Chicago, USA), ADH: quinone oxidoreductase (NQO‐1, 1:1000, ab28974, Abcam, Cambridge, MA, USA), Nrf‐2 (1:1000, 16396‐1‐AP, Proteintech, Chicago, USA), GAPDH (1:1000, 6000‐1‐lg, Proteintech, Chicago, USA), Histone H3 (1:1000, ab5013, Abcam, Cambridge, MA, USA), Fyn (1:1000, ab125016, Abcam, Cambridge, MA, USA), p‐AKT ser647 (1:1000,66444‐1‐lg, Proteintech, Chicago, USA) and p‐GSK3β ser9 (1:1000, #5558, CST, Danvers, MA, USA).

### 2.16 Statistical analysis

Two statistical methods including one‐way ANOVA test and Kruskal‐Wallis test were used. Firstly, we performed a normal distribution test for the data from different groups by Shapiro‐Wilk test. If *p *> 0.05, it indicated that the data complied with normal distribution. If *p *< 0.05, the data were not followed by normal distribution. If following the normal distribution, the one‐way ANOVA was used to analyse the statistical differences among the groups. *p *< 0.05 was considered the data with statistical difference, and *p *> 0.05 was no statistical difference. If not following the normal distribution, the Kruskal‐Wallis test was conducted, and *p *< 0.05 was regarded as statistical difference, and *p *> 0.05 was no statistical difference. Data were drawn from at least three independent experiments and were presented as the means ± SD. GraphPad Prism 8.02 (GraphPad Software, San Diego, CA, USA) was applied to analyse statistical significance.

## 3 RESULTS

### 3.1 Oridonin improved BBB integrity after ischaemic stroke

**FIGURE 1:**
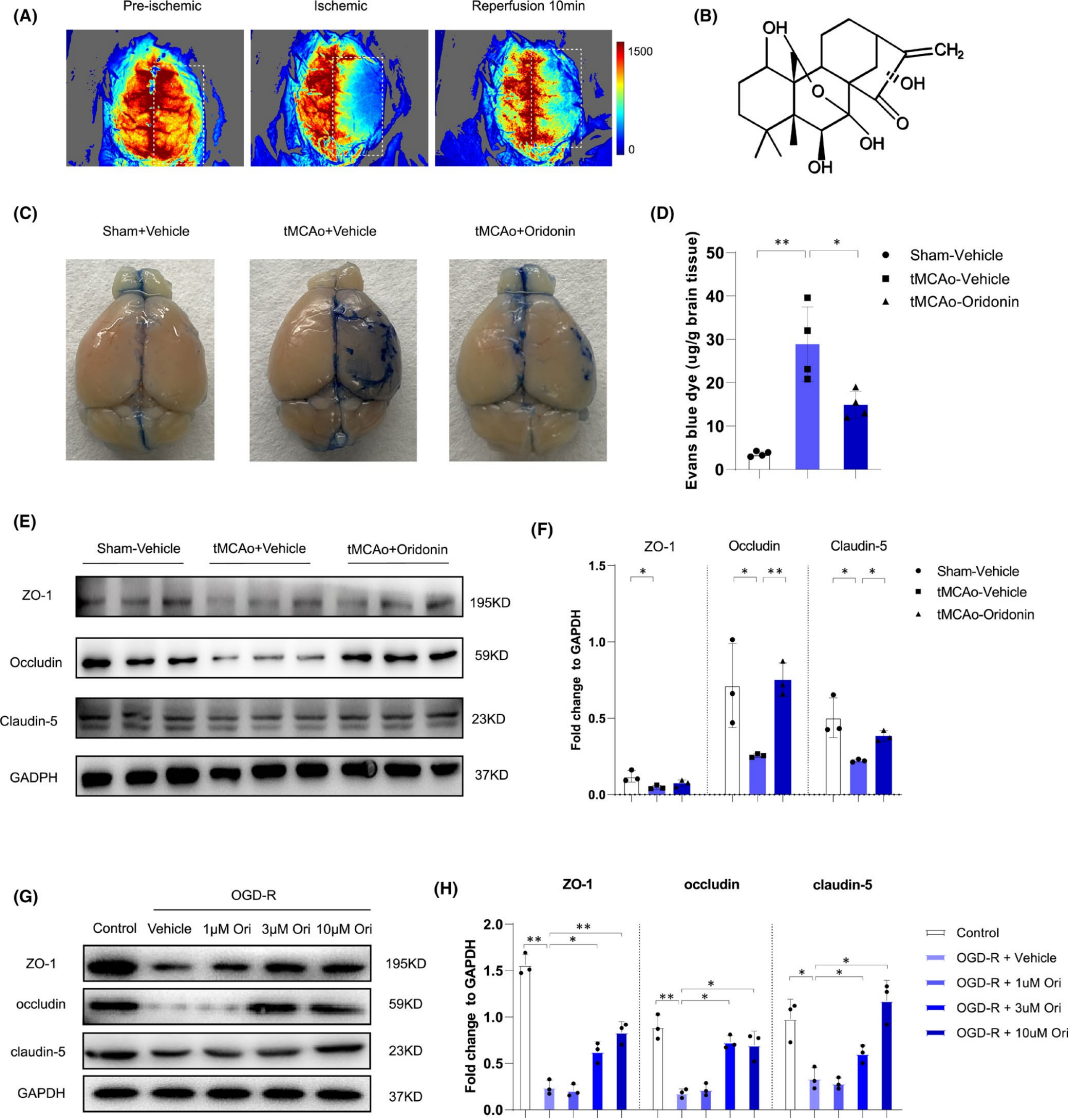
Oridonin inhibited the leakage of Evans blue to brain after ischaemic/reperfusion injury and increased TJ proteins in in vivo and in in vitro. (A) The images were captured from laser Doppler blood flow meter for verifying the model. (B) The molecular structure of Oridonin. (C and D) Representative images and quantification of EB leakage after tMCAO for 3 days in each group. The blue areas indicated the extravasation of EB (n = 4, data from different groups follow the normal distribution (Shapiro‐Wilk test, *p *> 0.5)). (E and F) Western blot analysis was performed in the ipsilateral brain after stroke for 3 days to detect the protein expression of ZO‐1, occludin and claudin‐5 (n = 3, data from different groups follow the normal distribution (Shapiro‐Wilk test, *p *> 0.5)). (G and H) Western blot analysis indicated oridonin significantly increased the TJ proteins expression of ZO‐1, occludin and accludin‐5 in bEND.3 cells after OGD/R (n = 3, data from different groups follow the normal distribution (Shapiro‐Wilk test, *p *> 0.5)). The one‐way ANOVA was used to analyse the statistical differences between the groups. The data are presented as mean ± SD, *p < 0.05, **p < 0.01

The tMCAO surgery was prepared in mice with the monitoring of laser Doppler blood flow meter (Figure 1A), and oridonin (Figure 1B) was administrated simultaneously when reperfusion. Reportedly, the downregulation of TJ proteins and impairment of BBB integrity are one of the major causes of ischaemic stroke injuries.^9^, ^10^ Accordingly, we first attempted to identify whether oridonin could alleviate the BBB disruption in ischaemic stroke mice. Through EB dye extravasation test, we found oridonin treatment effectively decreased EB dye extravasation in the ipsilateral cerebral hemisphere compared with tMCAO group treated with vehicle (Figure 1C,D). Next, we detected the expression of TJ proteins including occludin, claudin‐5 and ZO‐1 in the ipsilateral cerebral hemisphere. Our results found these proteins expression were all sharply decreased in tMCAO group. Significantly, oridonin treatment reversed such changes compared with tMCAO group alone (Figure 1E,F).

As is known, the brain vascular endothelial cells were the major component of BBB, which mainly expressed the TJ proteins.^6^, ^7^, ^8^ Thus, we further conducted in vitro experiments with bEND.3 cells and identified the specific effects after OGD/R and oridonin treatment. As shown in Figure 1G,H, oridonin treatment increased the expression of ZO‐1, occludin and claudin‐5 following OGD/R injury in a concentration‐dependent manner after OGD/R treatment.

### 3.2 Oridonin inhibited the infiltration of peripheral inflammatory cells and reduced the neuroinflammation

**FIGURE 2:**
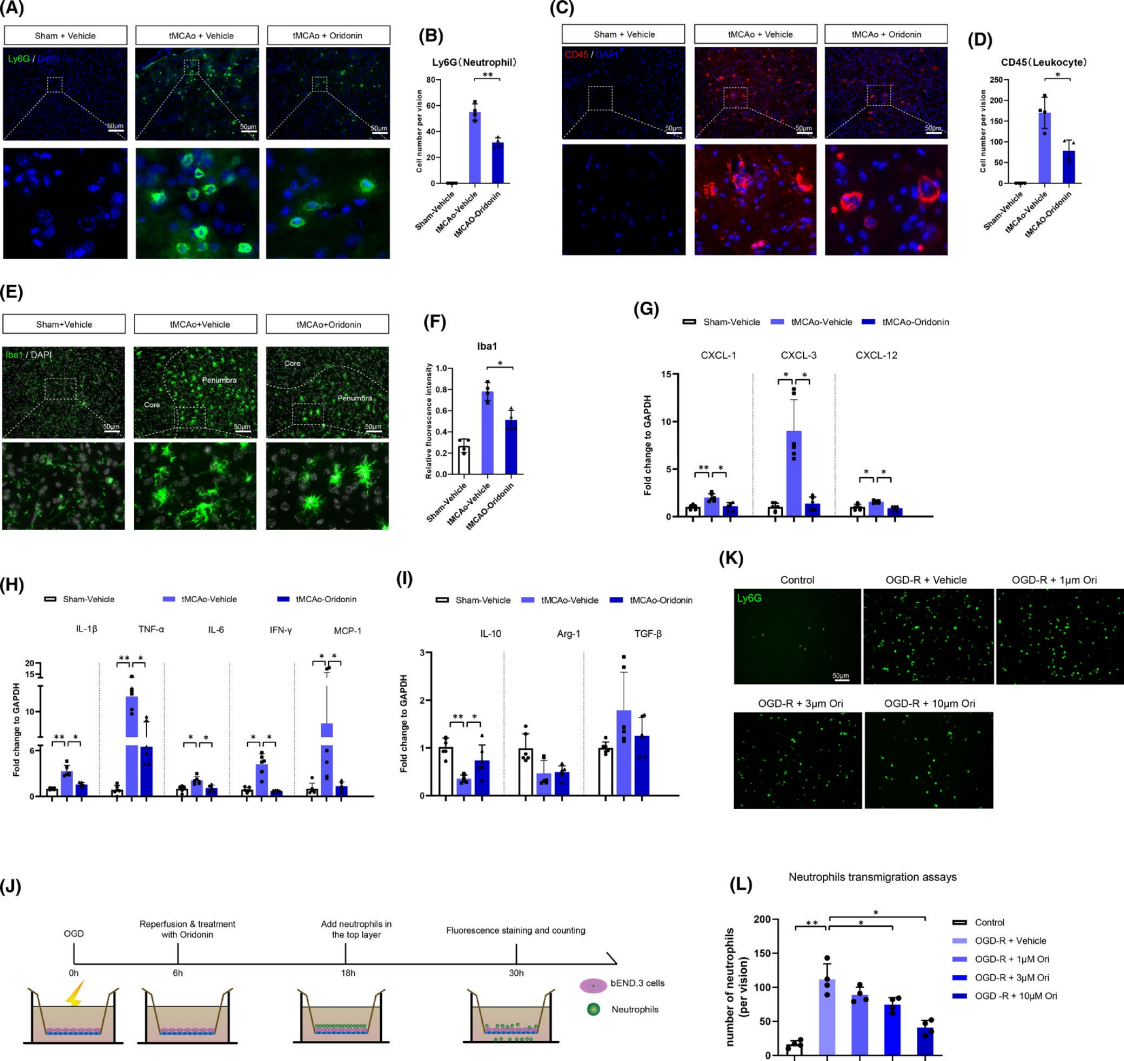
Oridonin prevented the infiltration of peripheral inflammatory cells to the penumbra region, decreased the chemokines mRNA levels and inhibited the activation of neuroinflammation in vivo and in vitro. (A–D) The number of inflammatory cells was examined by fluorescent staining at 72 h after tMCAO. The number of Ly6G^+^ neutrophils and CD45^+^ leukocytes was counted in each group (n = 4, data from different groups follow the normal distribution (Shapiro‐Wilk test, *p *> 0.5)). (E) Quantitative real‐time PCR was used to evaluate the mRNA expression of CXCL1 (n = 6, data from different groups follow the normal distribution (Shapiro‐Wilk test, *p *> 0.5)), CXCL3 (n = 6, data from different groups do not follow the normal distribution (Shapiro‐Wilk test, *p *< 0.5)) and CXCL12 (n = 6, data from different groups follow the normal distribution (Shapiro‐Wilk test, *p *> 0.5)). (F and G) Representative images and quantitative data of microglia in penumbra region of mouse at 72 h after tMCAO (n = 4, data from different groups follow the normal distribution (Shapiro‐Wilk test, *p *> 0.5)). (H and I) Quantitative real‐time PCR were used to determine the mRNA level of inflammatory factors, such as IL‐1β, TNF‐α, IL‐6, IFN‐γ, IL‐10, Arg‐1, TGF‐β (n = 6, data from different groups follow the normal distribution (Shapiro‐Wilk test, *p *> 0.5)) and MCP‐1 (n = 6, data from different groups do not follow the normal distribution (Shapiro‐Wilk test, *p *< 0.5)). (J) The pattern image of neutrophils infiltration tests with transwell experiment. (K and L) Cell fluorescence technology was used to check the number of neutrophils (Ly6G^+^) in the bottom layer (n = 4, data from different groups follow the normal distribution (Shapiro‐Wilk test, *p *> 0.5)). The one‐way ANOVA and Kruskal‐Wallis tests were used to analyse the statistical differences between the groups. Data are expressed as mean ± SD, *p < 0.05, **p < 0.01

The disrupted integrity of BBB led to the infiltration of peripheral inflammatory cells, thus aggravating neuroinflammation after ischaemic stroke.^5^ Basically, we observed the infiltration of neutrophils and leukocytes after oridonin treatment. Compared with tMCAO group, oridonin effectively decreased the number of Ly6G^+^ neutrophils (Figure 2A,B) and CD45^+^ leukocytes (Figure 2C,D) in the ipsilateral brain. Meanwhile, we measured the mRNA level of peripheral inflammatory cells infiltration related chemokines in the ipsilateral brain. Our data showed that oridonin treatment significantly reduced the mRNA expression level of CXCL1, CXCL3 and CXCL12 (Figure 2E). In addition, the activation of Iba1^+^ microglia (Figure 2F,G) was significantly decreased when compared with tMCAO group. Moreover, the mRNA expression level of pro‐inflammatory factors, including IL‐1β, TNF‐α, IL‐6, IFN‐γ and MCP‐1, was also markedly decreased after oridonin treatment (Figure 2H).  Compared with tMCAO group, the mRNA level of anti‐inflammation factor IL‐10 was obviously increased, whereas Arg‐1 and TGF‐β were not changed after oridonin treatment (Figure 2I).

**FIGURE 3:**
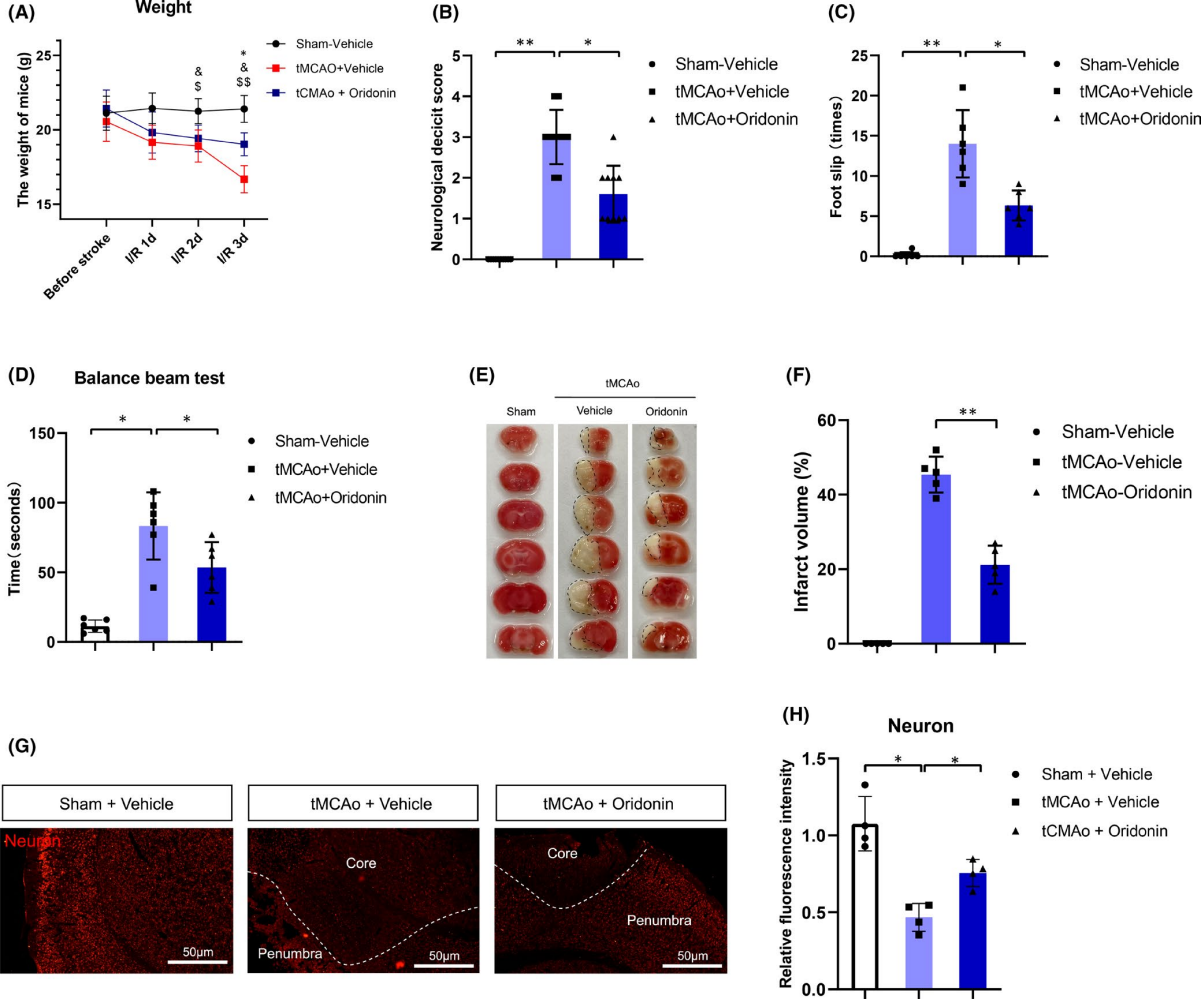
Oridonin improved neurological deficit score and reduced infract volume in mice subjected to tMCAO. (A) The body weight changes at 1d, 2d and 3d after tMCAO in each group. (B–D) Oridonin treatment improved the neurological deficit such as neurobiological deficit score, times of foot slip and balance beam hold time at 72h after tMCAO (n = 6–10, data from different groups follow the normal distribution (Shapiro‐Wilk test, *p *> 0.5)). (E and F) Oridonin treatment reduced cerebral infarct volume as evaluated by TTC staining (n = 5, data from different groups follow the normal distribution (Shapiro‐Wilk test, *p *> 0.5)). (G and H) NeuN+staining in the ipsilateral brain tissue and quantified by the fluorescent intensity (n = 4, data from different groups follow the normal distribution (Shapiro‐Wilk test, *p *> 0.5)). The one‐way ANOVA was used to analyse the statistical differences between the groups. Data are expressed as mean ± SD, *p < 0.05, **p < 0.01

To further confirm the effects of oridonin on the migration of neutrophils, we conducted the transwell system test with bEND.3 cells and neutrophils. As shown in Figure 3K,L, oridonin inhibited the migration of neutrophils after OGD/R concentration‐dependently. These results above confirmed that oridonin could inhibit the migration of inflammatory cells into brain after ischaemic stroke.

### 3.3 Oridonin improved neurological defects, reduced infarct volume and increased neurons survival in ischaemic mice

Next, we confirmed the curative effects on tMCAO mice mode of oridonin.

As shown in Figure 3A,B, oridonin treatment could significantly attenuate the weight loss and neurological behavioural defects in tMCAO model. In the balance beam test, we found the increase in foot slips and the time of duration were reversed by oridonin treatment (Figure 3C,D). Besides, compared with the vehicle group, oridonin markedly reduced the infarct volume by 25% after tMCAO (Figure 3E,F). Furthermore, the NeuN^+^ cells were significantly increased by oridonin treatment, which may contribute to the neurological recovery (Figure 3G,H).

### 3.4 Oridonin inhibited endothelial cells apoptosis both in vitro and in vivo

**FIGURE 4:**
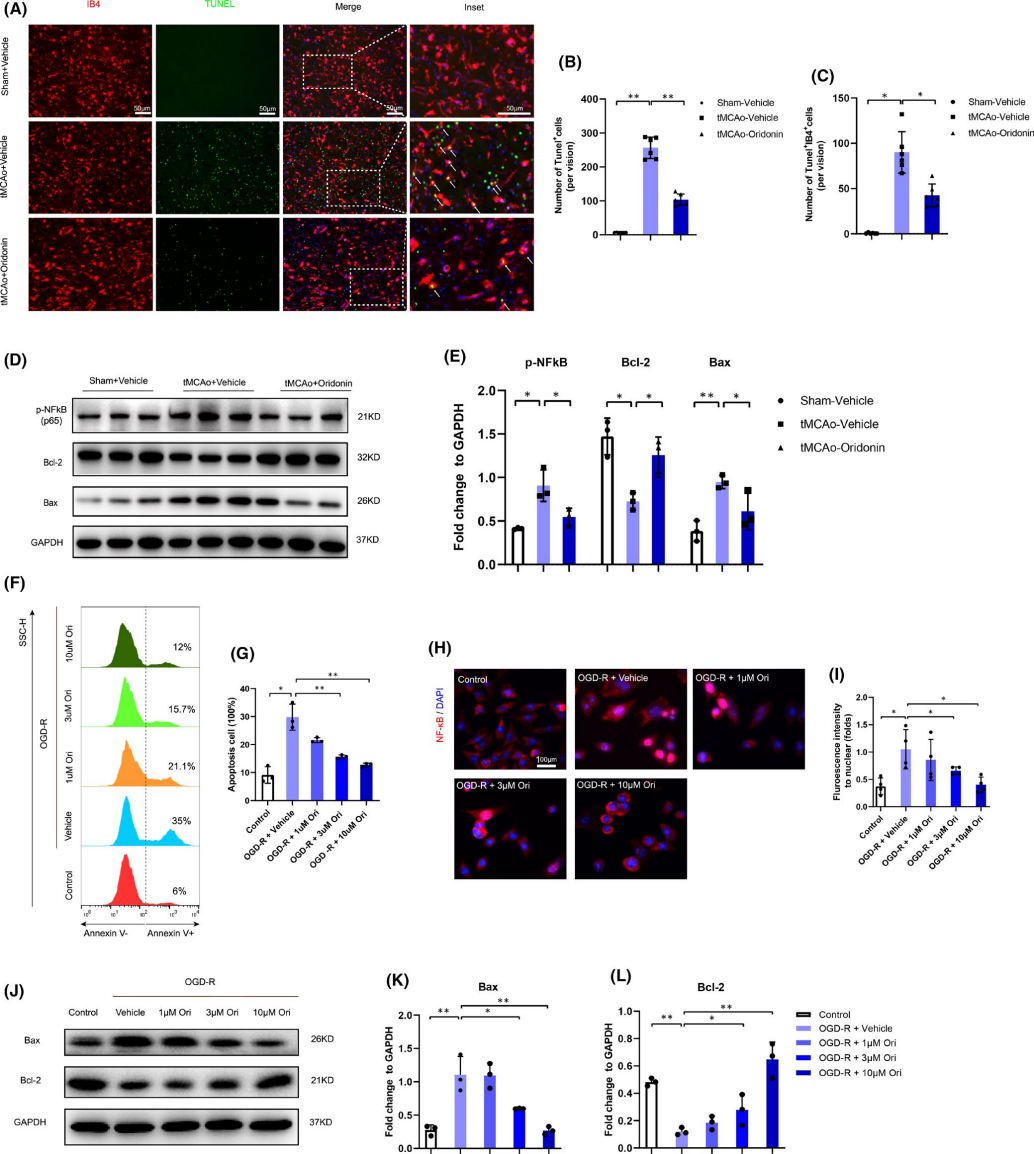
Oridonin treatment decreased endothelial cells apoptosis in vivo and in vitro. (A–C) The number of IB4^+^ endothelial cells and Tunel^+^ apoptotic cells were examined and counted by fluorescent staining at 72 h after tMCAO (n = 5 or 6, data from different groups follow the normal distribution (Shapiro‐Wilk test, *p *> 0.5)). (D and E) Western blot analysis was performed in the ipsilateral brain after stroke for 3 days to detect the protein expression of p‐NF κB, Bax and Bcl‐2 (n = 3, data from different groups follow the normal distribution (Shapiro‐Wilk test, *p *> 0.5)). (F and G) FACS analysis and a bar graph showed that oridonin decreased the ratio of Annexin V^+^ cells induced by OGD/R injury (n = 3, data from different groups follow the normal distribution (Shapiro‐Wilk test, *p *> 0.5)). (H and I) Immunofluorescent staining and fluorescence intensity analysis were performed for the translocation of NF‐κB from the cytosol to the nucleus (n = 4, data from different groups follow the normal distribution (Shapiro‐Wilk test, *p *> 0.5)). (J–L) Western blot was used to examine the expression of apoptosis‐related proteins Bcl‐2 and Bax in bEND.3 cells subjected to OGD/R with oridonin treatment (n = 3, data from different groups follow the normal distribution (Shapiro‐Wilk test, *p *> 0.5)). The one‐way ANOVA was used to analyse the statistical differences between the groups. The data are presented as mean ± SD. *p < 0.05, **p < 0.01

Endothelial cell apoptosis has been confirmed to be one of the fatal reasons for the decreased expression of tight junction proteins and the destruction of BBB integrity.^21^ As shown in Figure 4A, the increased number of TUNEL^+^ apoptosis cells and IB4^+^TUNEL^+^ apoptotic endothelial cells were reversed by oridonin treatment (Figure 4B,C) after tMCAO. Furthermore, the upregulation of pro‐apoptotic proteins NF‐κB (p65) and Bax, and the downregulation of anti‐apoptosis protein Bcl‐2 were also reversed by oridonin treatment after tMCAO (Figure 4D,E).

Flow cytometry results showed oridonin also significantly inhibited the apoptosis of endothelial cells in vitro in a concentration‐dependent manner (Figure 4F,G). It was reported the increase in NF‐κB (p65) translocation into the nucleus was closely associated with cell apoptosis. Immunofluorescence assay revealed that oridonin treatment attenuated NF‐κB translocation concentration‐dependently likewise (Figure 4H,I). Besides, Western blot analysis indicated that oridonin increased the expression of anti‐apoptotic factor Bcl‐2 (Figure 4J,K) and decreased the expression of pro‐apoptotic factor Bax (Figure 4 J and L).

These results above revealed that oridonin inhibited the apoptosis of endothelial cells after ischaemic stroke.

### 3.5 Oridonin promoted Nrf‐2 nuclear translocation via activating AKT(Ser473)/GSK3β(Ser9)/Fyn pathway and alleviated the oxidative stress‐induced injuries of endothelial cells

**FIGURE 5:**
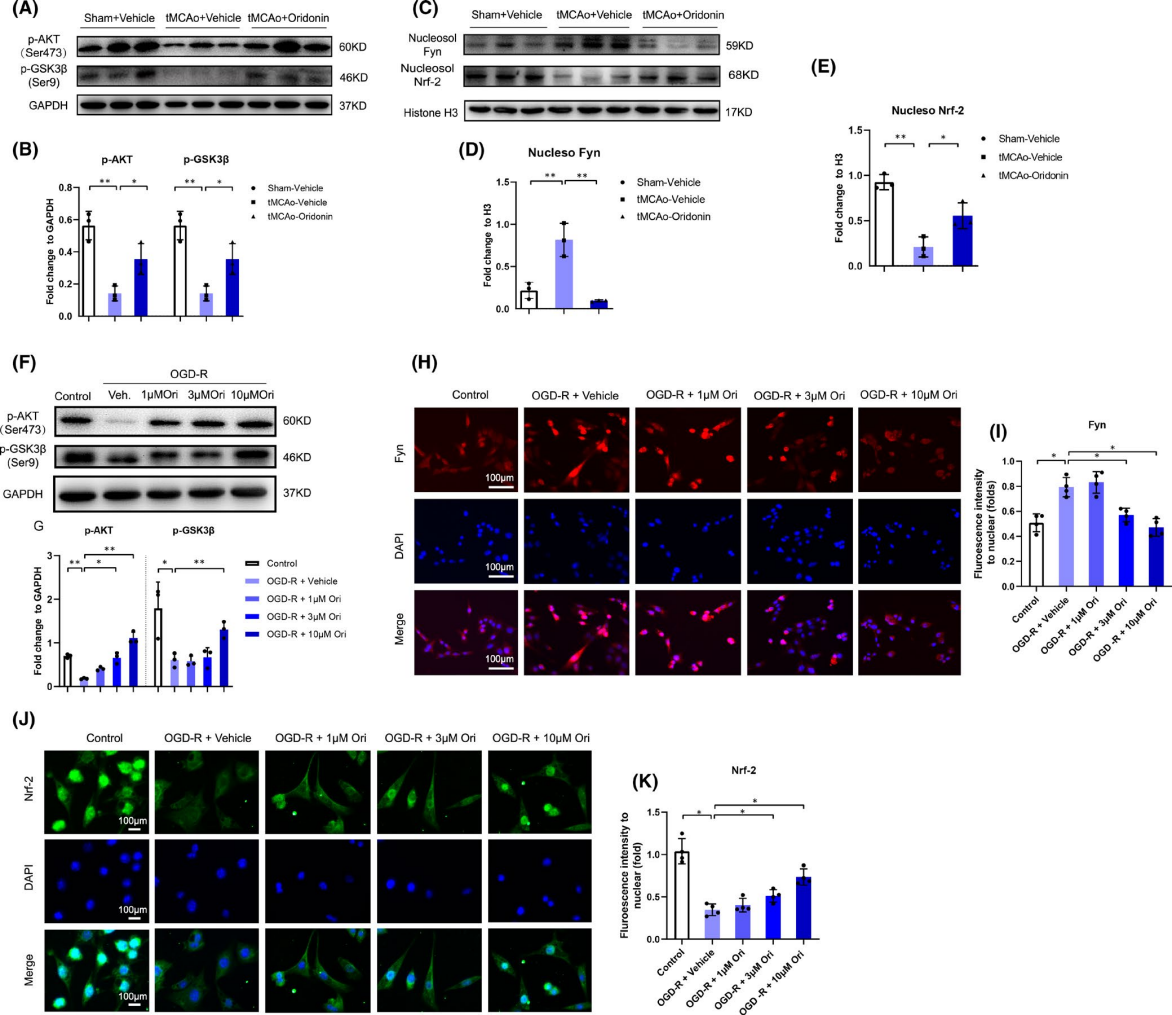
Oridonin activated the AKT(Ser473)/GSK3β(Ser9)/Fyn/Nrf‐2 signalling pathway in vivo and in vitro. (A and B) Western blot analysis was performed in the ipsilateral brain after stroke for 3 days to detect the protein expression of p‐AKT(Ser473) and p‐GSK3β(Ser9) expression (n = 3, data from different groups follow the normal distribution (Shapiro‐Wilk test, *p *> 0.5)). (C–E) Western blot analysis was performed in the ipsilateral brain after stroke for 3 days to detect the protein expression of nucleosol Fyn and nucleosol Nrf‐2. (n = 3, data from different groups follow the normal distribution (Shapiro‐Wilk test, *p *> 0.5)). (F and G) Western blot was used to examine the effects of oridonin on the p‐AKT(Ser473) and p‐GSK3β(Ser9) expression in bEND.3 cells (n = 3, data from different groups follow the normal distribution (Shapiro‐Wilk test, *p *> 0.5)). (H and I) Cell immunofluorescence was used to evaluate the effects of oridonin on regulating the nuclear translocation of Fyn in bEND.3 cells (n = 4, data from different groups follow the normal distribution (Shapiro‐Wilk test, *p *> 0.5)). (J and K) Cell immunofluorescence was used to evaluate the effects of oridonin on regulating the nuclear translocation of Nrf‐2 in bEND.3 cells (n = 4, data from different groups follow the normal distribution (Shapiro‐Wilk test, *p *> 0.5)). The one‐way ANOVA was used to analyse the statistical differences between the groups. The data are presented as mean ± SD. *p < 0.05, **p < 0.01

Numerous studies reported that severe oxidative stress reactions caused endothelial cells apoptosis and increased BBB permeability after ischaemic stroke.^22^, ^23^, ^24^ The Nrf‐2 signalling is one of the main regulators of oxidative stress reactions whose nuclear accumulation is closely associated with the balance of oxidative and anti‐oxidative system.^25^ The phosphorylation of tyrosine kinase Fyn translocating into nucleus can export the nuclear Nrf‐2 to cytosol for its ubiquitination and degradation, which could be regulated by AKT(Ser473)/GSK3β(Ser9) pathway.^26^ Notably, previous studies have found oridonin could regulate Nrf‐2 activity via promoting the phosphorylation of AKT(Ser473) in macrophages.^27^ In vivo, we found oridonin treatment increased the expression level of p‐AKT (Ser473) and p‐GSK3β (Ser9) in the ipsilateral brain (Figure 5A,B). In addition, oridonin decreased the protein expression of nuclear Fyn (Figure 5C,D) and increased the expression of nuclear Nrf‐2 (Figure 5 C and E). In bEND.3 cells, we also found oridonin promoted the phosphorylation of AKT(Ser473) and GSK3β(Ser9) at a concentration manner (Figure 5F,G) after OGD/R. Moreover, immunofluorescence suggested that oridonin decreased the Fyn nuclear translocation (Figure 5H,I) and increased the Nrf‐2 nuclear translocation (Figure 5J,K) concentration‐dependently after OGD/R.

**FIGURE 6:**
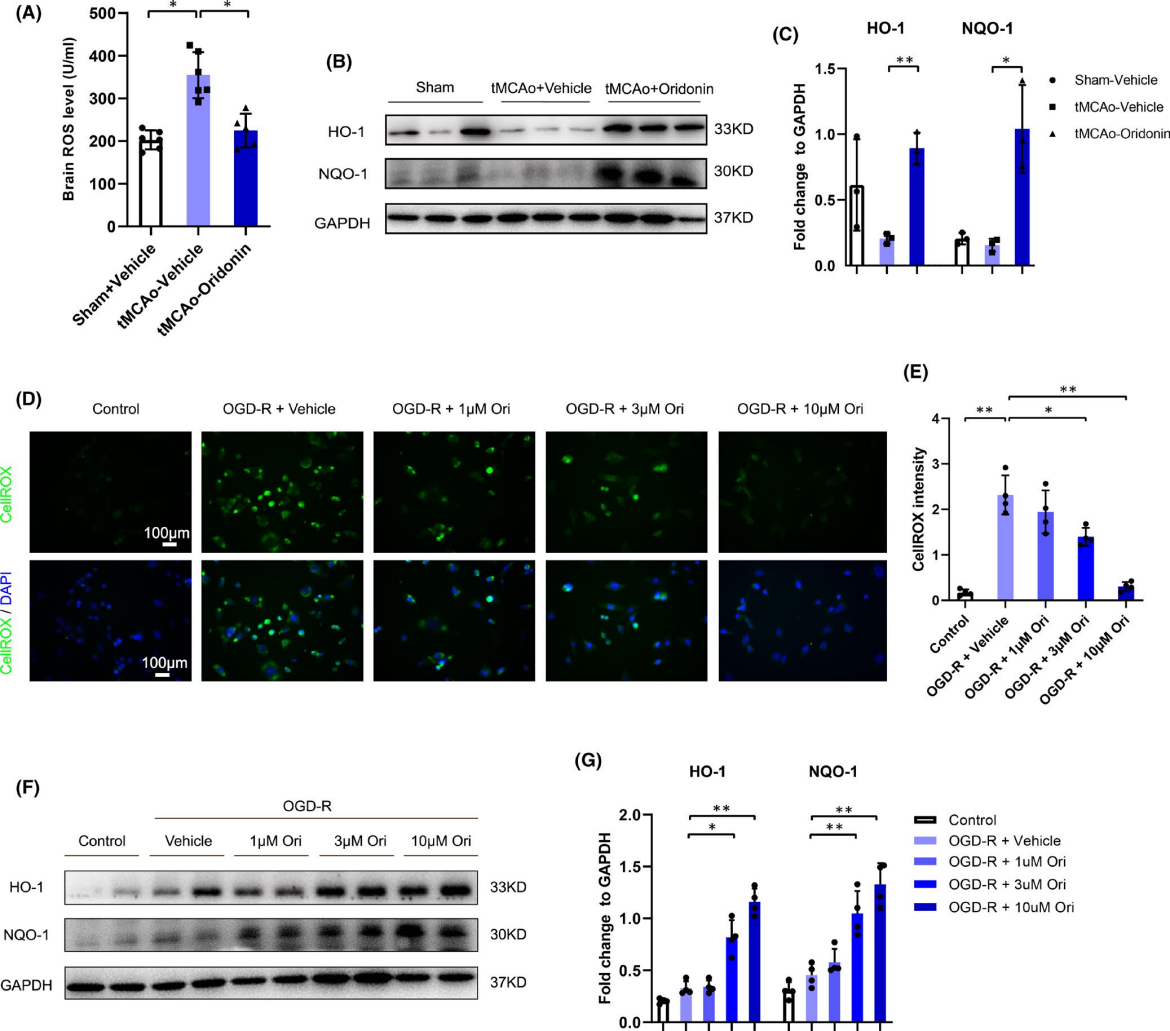
Oridonin inhibited ROS production, increased the expression of antioxidant enzymes in vivo and in vitro. (A) ROS level in the ipsilateral brain after stroke was measured by ROS Elisa assay in each group (n = 5 or 6, data from different groups follow the normal distribution (Shapiro‐Wilk test, *p *> 0.5)). (B and C) Western blot analysis was performed in the ipsilateral brain after stroke for 3 days to detect the protein expression of HO‐1 and NQO‐1. Quantification of the density of immunoreactive HO‐1 and NQO‐1 bands normalized to GAPDH in each group (n = 3, data from different groups follow the normal distribution (Shapiro‐Wilk test, *p *> 0.5)). (D and E) Effect of oridonin on OGD‐R induced intracellular ROS production was tested by CellROX assay (n = 4, data from different groups follow the normal distribution (Shapiro‐Wilk test, *p *> 0.5)). (F and G) Western blot was used to examine antioxidant enzymes HO‐1 and NQO‐1expression levels in bEND.3 cells subjected to OGD/R with oridonin treatment (n = 4, data from different groups follow the normal distribution (Shapiro‐Wilk test, *p *> 0.5)). The one‐way ANOVA was used to analyse the statistical differences between the groups. The data are presented as mean ± SD. *p < 0.05, **p < 0.01

Oxidative stress represented an imbalance between the production of ROS and the capacity of the antioxidant defence system.^28^ Moreover, mounting studies suggested oxidative stress could cause endothelial cell apoptosis and thus damage BBB integrity after stroke.^9^, ^10^ Thus, we next assessed whether oridonin could alleviate endothelial oxidative stress injury after ischaemic stroke. As shown in Figure 6A, ROS concentration displayed significant increase in the ipsilateral brain after tMCAO, whereas oridonin effectively suppressed ROS production. Meanwhile, the expression of anti‐oxidative enzymes HO‐1 and NQO‐1 in the ipsilateral was markedly increased (Figure 6B,C). Significantly, oridonin suppressed endothelial intracellular ROS production induced by OGD/R in a concentration‐dependently manner (Figure 6D,E). Oridonin upregulated HO‐1 and NQO‐1 expression concentration‐dependently likewise (Figure 6F,G). These data demonstrated that oridonin could protect endothelial cells from oxidative stress‐induced injuries after ischaemic stroke.

## 4 DISCUSSION

The homeostasis of the CNS is maintained by the BBB.^29^ Most neurological diseases, including stroke, lead to different degrees of BBB dysfunction.^30^, ^31^, ^32^, ^33^ Previous studies implied that neuroinflammation could be inhibited through improving the BBB integrity in animal models.^34^, ^35^, ^36^ Significantly, in our present study, we found EB dye extravasation in the ipsilateral cerebral hemisphere was reduced with oridonin treatment after ischaemic stroke. Endothelial cells are the major component of BBB and dominantly expressed TJ proteins. Our data showed that oridonin increased TJ proteins expression in both in vitro and in vivo. Accordingly, we identified that oridonin could exhibit protective effects in BBB integrity after ischaemic stroke.

Increasing evidence has shown that BBB destruction after ischaemic stroke indulged the infiltration of periphery inflammatory cells, such as neutrophils and leukocytes,^37^ which could aggravate neuroinflammation and exacerbate stroke outcome.^38^ Therefore, we observed the number of leukocytes and neutrophils in the ipsilateral hemisphere and found oridonin treatment significantly inhibited the infiltration of periphery inflammatory cells in the brain after ischaemic stroke. Additionally, the activation of Iba1+ microglia was reversed compared with vehicle‐treated tMCAO group after oridonin treatment. What is more, our results indicated the mRNA level of pro‐inflammatory factors was markedly decreased with oridonin treatment. These data suggested that oridonin inhibited the infiltration of inflammatory cells and alleviated neuroinflammation. We further confirmed the therapeutic effects of oridonin in ischaemic stroke mice by TTC test and neurological score test.

The apoptosis of endothelial cells directly caused the loss of TJ proteins and the disruption of BBB.^39^ Significantly, Licheng Gong etc. have found oridonin could relieve hypoxia‐induced apoptosis in H9C2 cells.^40^ Basically, we observed the protective effects of oridonin on endothelial cells apoptosis both in vivo and in vitro. The increased colocation of IB4^+^ endothelial cells and TUNEL^+^ apoptotic cells in vehicle‐treatment tMCAO group was reversed with oridonin treatment, and in vitro, FACS results of bEND.3 cells after OGD/R also showed oridonin significantly inhibited the cell apoptosis in a concentration‐dependent manner. Meanwhile, we detected the expression level of pro‐apoptotic proteins NF‐κB (p65) and Bax and anti‐apoptotic protein Bcl‐2. Consequently, oridonin could inhibit the apoptosis of endothelial cells.

Emerging reports suggest that Nrf‐2, as a vital nuclear transcriptional factor, shows strong anti‐oxidative activity and has been widely known as a promoter to inhibit oxidative stress and the resulting inflammation.^41^ Especially, the increase in Nrf‐2 nuclear translocation is strongly associated with anti‐oxidative stress injury via activating AKT(Ser473)/GSK3β(Ser473)/Fyn signalling pathway,^42^, ^43^ and reportedly, oridonin could exert protective effects on anti‐inflammatory and anti‐oxidative activities via modulating Nrf‐2.^14^ In our study, we found oridonin activated p‐AKT(Ser473) and p‐GSK3β(Ser473), decreased the nuclear translocation of Fyn and thereby increased the nuclear translocation of Nrf‐2, which were consistent with previous studies.^14^, ^35^, ^36^, ^37^ Additionally, oridonin treatment effectively increased the expression of anti‐oxidative enzyme HO‐1 and NQO‐1 and reduced the ROS level in the ipsilateral brain after tMCAO. These data further suggested that oridonin could promote the nuclear translocation of Nrf‐2 and improved the endothelial oxidative stress injury after ischaemic stroke.

## 5 CONCLUSIONS

**FIGURE 7:**
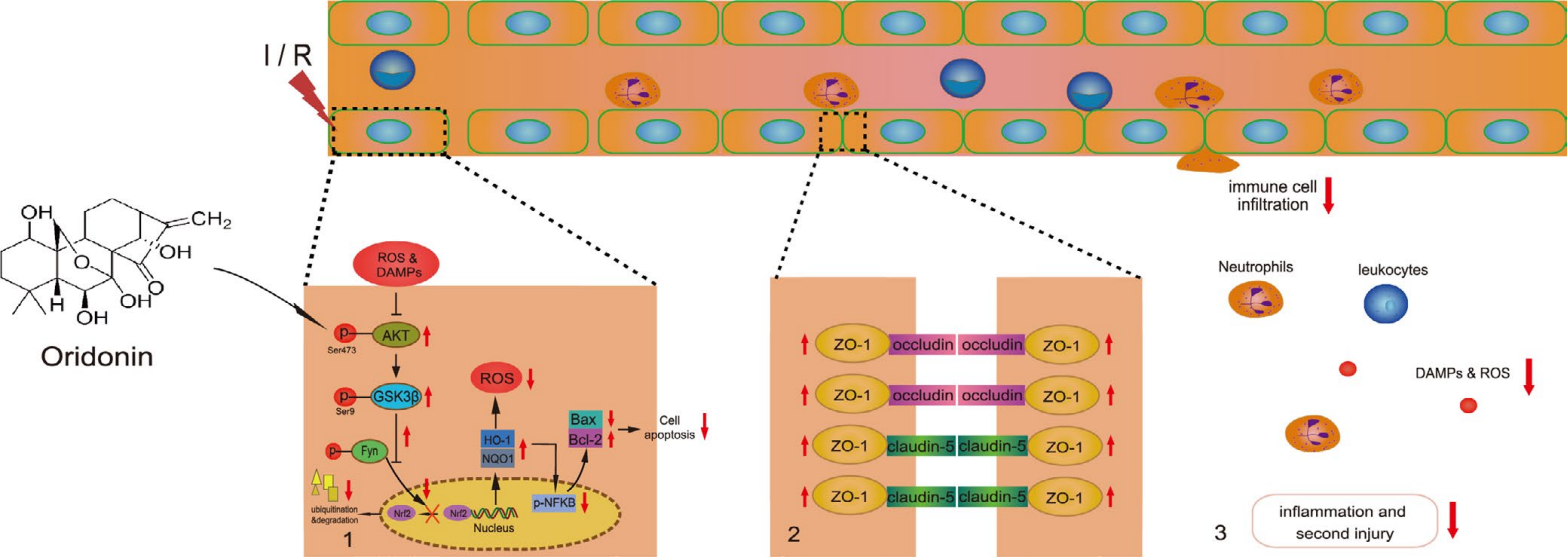
Mechanism diagram of oridonin‐mediated neuroprotective effects in ischaemic stroke. Oridonin activates the AKT/GSK3β/Fyn/Nrf‐2 signalling pathway and protects against oxidative stress injury in endothelial cells. Consequently, oridonin improves BBB integrity, ameliorates neuroinflammation and plays neuroprotection in ischaemic stroke

The present study revealed that oridonin improved BBB integrity and increased the TJ proteins expression via promoting the nuclear translocation of Nrf‐2 and inhibiting endothelial oxidative stress injury, and thereby prevented the infiltration of periphery inflammatory cells and reduced neuroinflammation after ischaemic stroke (Figure 7). Our findings provide fundamental evidence for the therapy of oridonin after ischaemic stroke.

## CONFLICT OF INTEREST

The authors declare no competing interests.

## AUTHOR CONTRIBUTION

**Lei Li:** Data curation (equal); Formal analysis (equal); Project administration (equal); Software (equal); Writing‐original draft (equal). **Shu‐Qi Cheng:** Data curation (equal); Formal analysis (equal); Methodology (equal); Software (equal); Writing‐review & editing (equal). **Wei Guo:** Formal analysis (equal); Project administration (equal); Software (equal); Writing‐original draft (equal). **Zhen‐Yu Cai:** Investigation (supporting); Methodology (equal). **Yu‐Qin Sun:** Formal analysis (equal); Software (supporting). **Xin‐Xin Huang:** Formal analysis (supporting); Writing‐original draft (supporting). **Jin Yang:** Formal analysis (supporting). **Juan Ji:** Software (supporting); Validation (supporting). **Ya‐yun Chen:** Data curation (supporting); Formal analysis (supporting). **Yin‐feng Dong:** Project administration (supporting); Software (supporting). **Hong Cheng:** Software (supporting). **Xiu‐Lan Sun:** Funding acquisition (lead); Investigation (lead); Resources (lead); Supervision (lead); Validation (lead); Writing‐original draft (equal).

## Data Availability

All data generated and analysed for this study are included in this published article.
